# LINE-1 methylation is inherited in familial testicular cancer kindreds

**DOI:** 10.1186/1471-2350-11-77

**Published:** 2010-05-17

**Authors:** Lisa Mirabello, Sharon A Savage, Larissa Korde, Shahinaz M Gadalla, Mark H Greene

**Affiliations:** 1 Clinical Genetics Branch, Division of Cancer Epidemiology and Genetics, National Cancer Institute, National Institutes of Health, Department of Health and Human Services, Bethesda, Maryland, USA; 2 Cancer Prevention Fellowship Program, National Cancer Institute, Bethesda, MD, USA

## Abstract

**Background:**

Testicular germ cell tumors (TGCT) are the most frequent cancers among young men. There is a clear familial component to TGCT etiology, but no high-penetrance susceptibility gene has been identified. Epigenetic aberrations of the genome represent an alternative mechanism for cancer susceptibility; and, studies suggest that epigenetic changes that influence cancer risk can be inherited through the germline. Global DNA hypomethylation has been associated with the risk of cancers of the bladder and head/neck.

**Methods:**

We performed a pilot study of global methylation at long interspersed nuclear elements-1 (LINE-1) in peripheral blood DNA isolated from 466 family members of 101 multiple-case testicular cancer families.

**Results:**

Investigating the correlation of LINE-1 methylation levels among parent-child pairs independent of affection status (*n *= 355) revealed a strong positive association only between mother-daughter (*r *= 0.48, *P *= <0.001) and father-daughter pairs (*r *= 0.31, *P *= 0.02), suggesting gender-specific inheritance of methylation. Incorporating cancer status, we observed a strong correlation in LINE-1 methylation levels only among affected father-affected son pairs (*r *= 0.49, *P *= 0.03). There was a marginally significant inverse association between lower LINE-1 methylation levels and increased TGCT risk, compared with healthy male relatives (*P *= 0.049).

**Conclusions:**

Our data suggest that heritability of LINE-1 methylation may be gender-specific. Further, the strong correlation between LINE-1 methylation levels among affected father-affected son pairs suggests that transgenerational inheritance of an epigenetic event may be associated with disease risk. Larger studies are needed to clarify these preliminary observations.

## Background

Testicular germ cell tumors (TGCT) are the most commonly diagnosed cancer among young American men aged 20-35 years, and their incidence has doubled over the last 40 years [1]. Family history of TGCT is one of the strongest and most consistent risk factors for this tumor. Brothers of affected cases have an 8-to 10-fold increased relative risk compared with the general population and fathers/sons a 4-and 6-fold higher risk [2,3]. These high familial risks suggest that inherited susceptibility and/or environmental factors that cluster in families may account for a significant portion of TGCT cases. Testicular microlithiasis (TM), a condition characterized by the presence of calcium deposits within the seminiferous tubules, aggregates in families and has been associated with testicular malignancy [4,5]. Linkage and candidate gene studies [6-10] have identified several genomic regions of interest, including Xq27, 2p23, 3p12, 3q26, 12p13-q21 and 18q21-q23. Candidate gene studies have identified two loci of interest, *i.e*., the Y-chromosome *gr/gr *deletion [9] and the *PDE11A *gene [8], while recent GWAS analyses identified *KIT-ligand*, *SPRY4 *and *BAK1*) [6,7]. However, a specific high-penetrance susceptibility gene has yet to be proven, suggesting that the combined contribution of multiple common genetic variants of lower penetrance may account for the inherited component of TGCT susceptibility [10].

Epigenetic changes in the genome, such as aberrant DNA methylation, are an increasingly recognized contributor to cancer development. TGCTs have distinct DNA methylation profiles, and parallels have been observed between the epigenetic properties of TGCTs and embryogenesis (*e.g*., DNA methylation increases with differentiation) [11]. The seminoma genome is essentially devoid of DNA methylation, while the nonseminoma genome is less extensively hypomethylated and has variable CpG island hypermethylation levels [12,13]. A few tumor suppressor genes are inactivated by DNA promoter hypermethylation in a small proportion of TGCTs, while DNA hypomethylation of testis-and cancer-associated genes and unmethylated *XIST *are frequently observed in TGCTs (Reviewed in [11]). Imprinting defects have also been observed in TGCT somatic tissues, suggesting that epigenetic defects may be present. In the aggregate, the data suggest that methylation may provide an alternate genetic mechanism for TGCT susceptibility.

Transgenerational epigenetic inheritance has been well studied and documented in many eukaryotic organisms (*e.g*., maize, yeast, Drosophila, mice) [14-16], and recent human studies suggest that epigenetic aberrations that influence cancer risk can be inherited through the germline from parent to child [17-20]. Epigenetic patterns have been observed to segregate in both Mendelian and non-Mendelian patterns, as well as in a pattern consistent with environmental exposure. Non-Mendelian and environmentally-induced transgenerational inheritance of epimutations is supported by model organism data [21-27]. There are several potential modes of transgenerational epigenetic inheritance, including paramutations, aberrant gene imprinting, and dsRNA processes (Reviewed in [28]). Retrotransposons are thought to be resistant to epigenetic reprogramming during embryogenesis in mice [29], and thus may play a role in epigenetic heritability (*e.g*., if inserted in or near the affected gene). A recent mouse model study found that transgenerational epigenetic inheritance through the germline controls susceptibility to TGCTs [21].

Global DNA hypomethylation, characterized by a global loss of 5-methylcytosine (5-mC), contributes to malignant transformation by activating oncogenes or latent retrotransposons, such as LINE-1, and by loss of imprinting [30]. LINE-1 retrotransposons are hypomethylated in many cancers and their lack of repression may contribute to genome disorganization, expression changes, and chromosomal instability [31]. The nucleotide patterns of LINE-1 repeats have been shown to both segregate in human pedigrees, and to provide an individual-specific fingerprint [32]. LINE-1 insertion dimorphisms may reflect DNA mutations or methylation changes [32]. Methylation levels at LINE-1 loci have been shown to differ among different loci in normal tissues, suggesting that *cis*-elements near each LINE-1 may influence their epigenetics [33]. In addition, locus-specific LINE-1 methylation has been shown to be differentially influenced by carcinogenic processes, depending on where the LINE-1s are located in the genome [33]. LINE-1 retrotransposons are strongly expressed in TGCTs [31,34,35], and it was hypothesized that active LINE-1 retrotransposon events are involved in primordial germ cell differentiation, and the consequential genome instability contributes to malignant transformation [35].

In recent case-control studies, hypomethylation of peripheral blood DNA was found to be associated with increased risks of bladder and head/neck cancers, suggesting that global demethylation in genomic DNA is a potential biomarker of cancer susceptibility [36,37]. We performed a pilot analysis of global DNA methylation levels in peripheral blood among family members from 101 multiple-case familiar testicular cancer families. Methylation at LINE-1 regions using pyrosequencing was used as a proxy for measuring global methylation levels. We examined the heritability of the global genomic methylation phenotype in our families, as well as the association between global (LINE-1) methylation levels and testicular cancer.

## Results

DNA from 152 patients with TGCT and 314 unaffected family members from 101 multiple-case testicular cancer families were available for LINE-1 methylation analysis. Table 1 shows the characteristics of the 466 study participants. Males had significantly higher levels of LINE-1 methylation than females (*P *= 0.002). There were no significant effects of age, smoking, or alcohol consumption on LINE-1 methylation levels (Additional file 1). There was no significant difference in mean LINE-1 methylation between men with and without microlithiasis (*P *= 0.608).

**Table 1:** Global (LINE-1) methylation levels and characteristics of all subjects.

Variables	No. (%)	Mean level*	95% CI	***P***
Family, healthy	255 (54.7)	79.31	79.06-79.56	
Family, other cancer	39 (8.4)	79.11	78.47-79.74	
Family, other condition^§^	20 (4.3)	79.71	78.84-80.58	
TGCT patients	152 (32.6)	79.13	78.78-79.45	0.377^¶^
Multi-case families	118 (77.6)	79.50	79.12-79.88	
Sporadic bilateral TGCT	34 (22.4)	78.91	78.21-79.60	0.142^¥^
Gender^‡^				
Male	114 (44.7)	79.60	79.26-79.95	
Female	141 (55.3)	78.87	78.55-79.18	**0.002^†^**
Patient TGCT type				
NOS	4 (2.6)	77.74	75.68-79.81	
Seminoma	73 (48)	79.19	78.70-79.68	
Non-seminoma	75 (49.3)	79.59	79.11-80.08	0.267^a^
Choriocarcinoma	2 (2.7)	79.53	76.86-82.20	
Embryonal	15 (20)	79.88	78.90-80.86	
Germinoma	2 (2.7)	78.58	75.91-81.26	
Mixed germ cell	34 (45.3)	79.19	78.52-79.86	
Teratocarcinoma	20 (26.7)	80.78	79.84-81.73	
Yolk sac tumor	2 (2.7)	76.06	74.27-79.85	

Total participants	466	79.25	79.08-79.43	

* Adjusted for age;

^§ ^Other testicular conditions (*e.g*., hernia or cryptorchidism);

^‡ ^Healthy family only;

^¶ ^For methylation level difference between TGCT patients and healthy family, adjusted for age and sex;

^¥ ^For methylation level difference between patients from multiple case families *vs*. sporadic bilateral TGCT patients, adjusted for age;

^† ^For methylation level difference between males and females, adjusted for age;

^a ^For methylation level difference between TGCT patients with seminoma *vs*. non-seminoma, adjusted for age;

NOS = not otherwise specified.

### Inheritance of global (LINE-1) methylation

**Figure 1:**
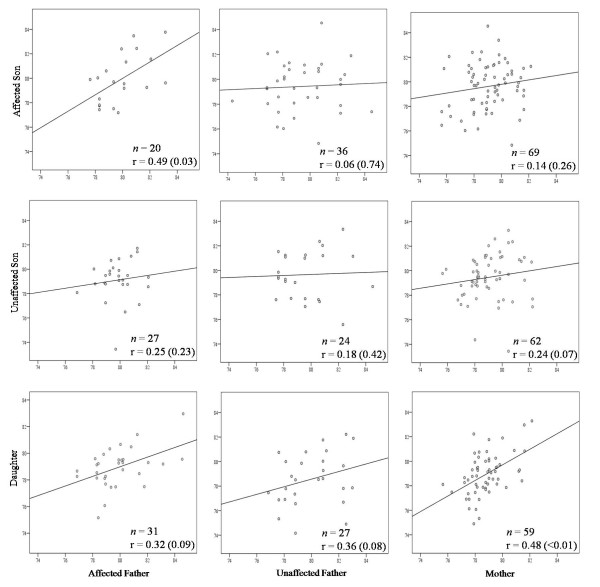
**Familial relations of global (LINE-1) methylation levels by patient status**. Each plot shows the number of pairs (*n*), correlation coefficient (r) and corresponding *P *value. Affected refers to patients with cancer, and unaffecteds are cancer-free.

We assessed whether LINE-1 methylation levels in offspring were correlated with either paternal or maternal levels (*n *= 355 pairs), irrespective of patient status (Table 2). These analyses revealed a high correlation in LINE-1 methylation between mother-daughter (*r *= 0.48, *P *= 0.0002; Figure 1) and father-daughter pairs (*r *= 0.31, *P *= 0.021), but only a weak correlation between mother-son (*r *= 0.18; *P *= 0.047) and father-son pairs (*r = *0.16; *P *= 0.114).

**Table 2:** Intra-familial correlations adjusted for age.

Relationship	No. of pairs	*r*^†^	*P *value
Parent-children	355	0.228	**<0.0001**
Mother-children	190	0.271	**0.0002**
Mother-daughter	59	0.476	**0.0002**
Mother-son	131	0.176	**0.047**
Father-children	165	0.187	**0.018**
Father-daughter	58	0.308	**0.022**
Father-son	107	0.157	0.114

^†^Spearman rank correlation coefficient adjusted for parental age, offspring age, and disease status of the males.

Figure 1 illustrates LINE-1 methylation level correlations between parent-child pairs after stratifying by testicular cancer status. There was a statistically significant correlation in LINE-1 methylation levels between affected father-affected son pairs (*r *= 0.49, *P *= 0.034). Affected father methylation levels were not significantly correlated with unaffected son levels (*P *= 0.228). Furthermore, when comparing unaffected father-and mother-affected son pairs, no significant associations were demonstrated (*r *= 0.06, *P *= 0.738, and *r *= 0.14, *P *= 0.257, respectively). There were no significant differences between the correlations among unaffected sons and daughters with affected and unaffected fathers (Figure 1).

### Patients with testicular germ cell tumors and global (LINE-1) methylation

As previously shown, younger age (26-50 years of age) and the presence of testicular microlithiasis were each significantly associated with testicular cancer risk (independent of methylation levels; Table 3). There was no statistically significant difference between LINE-1 methylation levels in TGCT patients and healthy male family members (patient mean: 79.1 [95% CI 78.8-79.5] *versus *healthy male mean: = 79.6 [95% CI 79.3-79.9]; *P *= 0.297). A comparison of LINE-1 methylation as a continuous variable yielded a marginally statistically significant association between decreasing LINE-1 methylation level and testicular cancer (OR per unit change = 1.15, 95% CI 1.00-1.32; *P *= 0.049). Males subjects were categorized into tertiles of LINE-1 methylation level based on the methylation distribution among healthy male family members. An upward trend, not statistically significant, was observed when comparing the lowest and middle tertiles to the highest (OR = 1.41 [95% CI 0.71-2.80] and 1.83 [95% CI 0.91-3.66], respectively; *P*_trend _= 0.089) (Table 4). Similarly, with LINE-1 methylation dichotomized at the median in healthy male controls, lower methylation was associated with a non-significant increase in testicular cancer risk (OR = 1.48, 95% CI 0.85-2.55).

**Table 3:** Association of individual variables with patient status.

Variables	Healthy Males		TGCT Cases		OR^† ^(95% CI)
			
	No.	(%)	No.	(%)	
Age:					
0-25	27	(23.68)	11	(7.28)	1.00 (ref)
26-50	34	(29.82)	108	(71.52)	**8.30 (3.62, 19.05)**
51-75	47	(41.23)	31	(20.53)	1.72 (0.72, 4.10)
76-100	6	(5.26)	1	(0.66)	0.52 (0.05, 4.92)
Alcohol use:					
Never	25	(30.86)	39	(26.90)	1.00 (ref)
Former	14	(17.28)	33	(22.76)	1.12 (0.45, 2.77)
Current	42	(51.85)	73	(50.34)	0.78 (0.38, 1.64)
Smoking status:					
Never	41	(50.00)	97	(66.44)	1.00 (ref)
Former	28	(34.15)	38	(26.03)	0.67 (0.33, 1.35)
Current	13	(15.85)	11	(7.53)	0.42 (0.16, 1.14)
Microlithiasis					
No	23	(88.46)	32	(59.26)	1.00 (ref)
Yes	3	(11.54)	22	(40.74)	**5.69 (1.41, 23.04)**

^† ^Odds ratios (95% confidence intervals) adjusted for age and/or microlithiasis status.

**Table 4:** Association of TGCT with blood-derived DNA global (LINE-1) methylation levels.

Relative methylation level in tertiles	Healthy Males^§^	TGCT Cases*	OR^† ^(95% CI)	*P *value
				
	No. (%)	No. (%)		
All patients				
Low (≤78.8)	38 (33.3)	57 (37.8)	1.83 (0.91, 3.66)	0.089
Middle (78.9-80.6)	39 (34.2)	52 (34.4)	1.41 (0.71, 2.80)	
High (>80.6)	37 (32.5)	42 (27.8)	1.00 (ref)	
Patients with seminoma				
Low	38 (33.3)	29 (39.7)	2.21 (0.90, 5.43)	0.085
Middle	39 (34.2)	27 (37.0)	1.76 (0.72, 4.30)	
High	37 (32.5)	17 (23.3)	1.00 (ref)	
Patients with non-seminoma				
Low	38 (33.3)	26 (35.1)	1.56 (0.67, 3.66)	0.306
Middle	39 (34.2)	23 (31.5)	1.18 (0.50, 2.78)	
High	37 (32.5)	25 (33.8)	1.00 (ref)	
Multi-case families^‡^				
Low	38 (33.3)	41 (35.3)	1.68 (0.80, 3.49)	0.174
Middle	39 (34.2)	41 (35.3)	1.51 (0.74, 3.10)	
High	37 (32.5)	34 (29.3)	1.00 (ref)	
Bilateral TGCT families^¶^				
Low	38 (33.3)	15 (44.1)	2.12 (0.75, 6.01)	0.218
Middle	39 (34.2)	11 (32.4)	1.23 (0.43, 3.46)	
High	37 (32.5)	8 (23.4)	1.00 (ref)	

^† ^Odds ratios (95% confidence intervals) adjusted for age and microlithiasis status;

^§ ^Males without cancer or other testicular abnormalities;

* Only 151 total TGCT cases, one subject with teratocarcinoma failed bisulfite conversion;

^‡ ^Families with two or more cases of TGCT;

^¶ ^A single family member had bilateral testicular cancer.

Associations between lower LINE-1 methylation and TGCT risk were stronger in patients with seminoma *versus *non-seminomatous tumors, and in bilateral TGCT patients *versus *patients from multi-case families (Table 4). We also examined the association between TGCT and LINE-1 methylation after stratification by age (data not shown). For those < 50 years, the OR for the lowest LINE-1 methylation level (1^st ^tertile) was 1.61 (95% CI 0.57-2.59), *versus *1.48 (95% CI 0.48-4.60) for those ≥50.

## Discussion

We found that males had significantly higher global (LINE-1) methylation levels than females, consistent with prior reports [37-39]. This difference has been variably attributed to gender-related dietary differences, reduced circulating levels of folate due to female menstruation, and/or the presence of an additional X chromosome in female cells [37,39]. We found no association between LINE-1 methylation levels and age [36,37,40], smoking [36,38], or alcohol usage [36,37], also consistent with prior studies.

LINE-1 methylation levels of offspring were found to be significantly positively correlated with parental levels, particularly between mother-daughter, father-daughter, and affected father-affected son pairs. There are limited data investigating epigenetic inheritance in humans, although it has been suggested that epimutations may be maintained more frequently during oogenesis [41,42] than during spermatogenesis [43]. Differences in epigenetic reprogramming between maternal and paternal genomes have been observed in mouse and drosophila studies [22,23,25-27]. In mice, epigenetic inheritance at a locus involved in fur color demonstrated that there was incomplete erasure of an epigenetic modification at a retrotransposon when passed through the female germ line [23].

However, others have shown that exposure to an endocrine disruptor during embryonic gonadal sex determination in rats induced permanent DNA methylation imprinting of the male germline (*i.e*., sperm) that was transmitted to subsequent generations [25-27]. This epigenetic alteration was associated with transgenerational disease states (approximately 85% frequency), including abnormalities in testis function, male infertility, and tumor development. This observation illustrates how an environmental exposure can epigenetically reprogram the germline and promote adult-onset defects in the germline, providing one possible mechanism by which environmental exposures may promote TGCTs [44].

Transgenerational epigenetic interactions have been shown to control susceptibility to TGCTs in mice with *Dead-end homologue 1 *(*Dnd1*) mutations [21]. It was suggested that these epigenetic modifiers may mark the germline, and offspring that also carry a genetic susceptibility factor will have an increased risk of TGCTs; or, environmental factors may influence genes to act as mediators and to monitor cellular conditions that then induce epigenetic alterations [28]. Lam *et al. *[21] suggested that, in humans, transgenerational epigenetics may explain the 2-3 fold risk difference in brothers and sons of TGCT affected individuals. The strong correlation we observed between LINE-1 methylation levels among affected father-affected son pairs may reflect an inherited or germline LINE-1 hypomethylation abnormality. Such an inherited epigenetic event could be associated either with an environmental factor or genetic susceptibility.

We observed an association between TGCT and global hypomethylation that was marginally significant when considering LINE-1 methylation as a continuous variable after controlling for potential confounders and testicular cancer risk factors (age and microlithiasis). Our study had ≥80% statistical power to detect ORs of 1.6 and 2.4 with LINE-1 methylation in the second and first tertiles, respectively, assuming a type I error of 0.05. Other studies have found that global hypomethylation in DNA from blood was associated with bladder and head/neck cancers [36,37]. Genomic global (LINE-1) methylation may be an independent risk factor or a phenotypic marker of cancer risk associated with genetic instability, aberrant epigenetic regulation, or other factors.

Our small sample size with attendant reduced statistical power to detect differences in LINE-1 methylation levels is a limitation of this study, although ours is the largest cohort of multiple-case families available for analyses of this kind. The family-based design of this study also limits our exploring the association between TGCT and global hypomethylation levels, since we have observed that *this may be an heritable *phenotype; a non-family based matched control group would be a useful follow-up. However, the unique multiple-case family design of this study is a strength for investigating inheritance, which allowed us to study multiple individuals per family with and without cancer, and to eliminate potential confounding due to ethnic background or population stratification.

LINE-1 retrotransposons are highly-activated in TGCTs and may lead to insertional mutations, transcriptional deregulation, DNA breaks, and an increased frequency of recombinational events [31,34,35]. Little is known regarding the mechanisms underlying hypomethylation changes of LINE-1 sequences; however, available data suggest that LINE-1 retrotransposon events may contribute to genome instability and malignant transformation. This is the first study to show that global LINE-1 methylation levels may be inherited from parent to child in humans, and that hypomethylation is associated with testicular cancer risk. More research is needed to help explain differences in maternal/paternal effects and mechanisms of transgenerational epigenetic inheritance.

## Conclusions

The results of this study suggest that LINE-1 methylation is heritable in humans, and that transgenerational inheritance of LINE-1 methylation levels may be associated with testicular cancer risk. The incomplete penetrance and non-Mendelian inheritance of epigenetic variation are consistent with complex disease phenotypes, which familial testicular cancer displays, and thus it may provide some insight into the basis of this complex disease. It has proved difficult to identify high-penetrance TGCT susceptibility genes in humans, despite a high familial risk and likely inherited susceptibility. Transgenerational epigenetic mechanisms could help explain the difficulty of finding classical TGCT susceptibility genes in humans. Larger studies are warranted to further explore the association between genomic global hypomethylation and TGCT risk, and to investigate the hypothesis that LINE-1 epigenetic inheritance is associated with TGCT susceptibility.

## Methods

### Study population

Families with two or more cases of TGCT or a single family member with bilateral testicular cancer were ascertained as part of the NCI Clinical Genetics Branch Familial TGCT study, details of which are described elsewhere [4]. Men with sporadic bilateral TGCT were included because of the known excess risk of bilateral affection in familial TGCT [45]. In brief, "cases" were men with histologically-confirmed cancer of one or both testes. Family members were invited to participate in the study if they were first-degree relatives of a case (aged ≥12yrs), spouses of a case who had participating children, non-first-degree blood relatives who provided a genetic link between cases, or blood relatives with cancer. All participants completed detailed questionnaires of medical and family history and risk factors, provided blood samples, and a subset of participants underwent a detailed medical evaluation at the NIH Clinical Center. Blood was collected between 2003 and 2006 and stored at the NCI/CGB Biorepository. The majority of patients underwent blood collection 5 or more years after they were diagnosed with TGCT (mean time between diagnosis and blood draw was 9 yrs; median time = 5 yrs). We observed no significant correlation between number of years from cancer diagnosis/treatment to study blood draw and methylation level (*P *= 0.492; data not shown). The parent study has been reviewed and approved by the NCI Institutional Review Board (NCI Protocol 02-C-0178; NCT00039598), and all participants provided written informed consent.

### DNA extraction and bisulfite treatment

Genomic DNA was extracted from fresh whole blood by standard methods. For bisulfite conversion, DNA was treated using EZ DNA Methylation-Gold™ Kit (Zymo Research Corp., CA, USA) according to the manufacturer's recommendations. The final elution was performed with 30 μl of M-Elution Buffer.

### LINE-1 PCR and pyrosequencing

DNA methylation (%5-mC) of LINE-1 was quantified using PCR-pyrosequencing of the bisulfite-treated DNA by EpigenDx Laboratory Service (Worcester, MA), as previously described [46]. In brief, the bisulfite-treated DNA was amplified by PCR using primers designed toward a consensus LINE-1 sequence. A 50-μL PCR was carried out in 25-μL GoTaq Green Master mix (Promega, Madison, WI, USA), 1 pmol of the forward primer (TTTTGAGTTAGGTGTGGGATATA), 1 pmol of the biotinylated reverse primer (biotin-AAAATCAAAAAATTCCCTTTC), 50 ng of bisulfite-treated genomic DNA, and water. Biotin-labeled primers were used to purify the final PCR product using Sepharose beads. The PCR product was bound to Streptavidin Sepharose HP (Amersham Biosciences, Uppsala, Sweden) and the Sepharose beads containing the immobilized PCR product were purified, washed, and denatured using a 0.2 M NaOH, and washed again using the Pyrosequencing Vacuum Prep Tool (Pyrosequencing, Inc., Westborough, MA, USA), as recommended by the manufacturer. Then, 0.3 μM of the pyrosequencing primer was annealed to the purified single-stranded PCR product and pyrosequencing was performed using the PSQ-HS 96 Pyrosequencing System (Pyrosequencing, Inc.). The relative 5-mC content was expressed as percentage of methylated cytosines divided by the sum of methylated and unmethylated cytosines (5-mC/[5-mC + unmethylated cytosine] = %5-mC). Built-in controls were used to verify bisulphite conversion efficiency. To increase precision, each sample was tested four times for LINE-1 methylation, and the 4-test mean was used in statistical analyses. The coefficient of variation (CV) among 48 blinded replicate samples was 5.7%, and the inter-plate CV was 2.9%.

### Statistical analysis

We tested the intra-familial (parent-offspring pairs) correlation of LINE-1 methylation using Spearman rank correlations. Scatterplots were also used to explore the relationship between LINE-1 methylation levels and parent-child pairs. In this analysis, male participants were stratified according to their testicular cancer status. These correlations were adjusted for parental and offspring age, and the un-stratified analyses were additionally adjusted for case status. Spearman rank correlations and general linear models were used to investigate the strength of the associations between %5-mC and age, smoking status (never, current, former), and alcohol consumption (never, current, former). We adjusted for age in all analyses, since age has been associated with global hypomethylation [47,48]. For microlithiasis, only men who have had a testicular ultrasound were included in the analysis as lacking microlithiasis. We used linear regression models to evaluate potential predictors of LINE-1 methylation levels, as a continuous variable. To evaluate the association between TGCT and LINE-1 methylation, we used unconditional logistic regression models to calculate the odds ratio (OR) and 95% confidence intervals (CI) adjusting for potential confounders (*e.g*., age). LINE-1 methylation levels were categorized in tertiles and dichotomized at the median, based on the distribution in the healthy male family controls; females were excluded from these analyses. We computed the variance of the OR estimates using a robust variance estimator [49] to adjust for the correlations between participants from the same family. Covariate data were taken from study questionnaires, physician history and physical exams. All tests were two-sided. Statistical significance refers to a *P *≤ 0.05 or an OR 95% CI that excludes 1.0. All analyses were carried out using SAS software version 9.1 (SAS Institute, Cary, NC).

## Competing interests

The authors declare that they have no competing interests.

## Authors' contributions

LM designed the study, performed the methylation and statistical analyses, and drafted the manuscript. SAS helped design the study, participated in statistical analyses and manuscript preparation. LK performed medical evaluation of patients, case management and patient/family data collection. SMG helped with the statistical analysis accounting for relatedness. MHG designed the Familial Testicular Cancer study, and provided critical input to the current analysis strategy, access to resources, and manuscript editing. All authors read and approved the final manuscript.

## Pre-publication history

The pre-publication history for this paper can be accessed here:

http://www.biomedcentral.com/1471-2350/11/77/prepub

## Supplementary Material

Additional file 1 **Global (LINE-1) methylation levels among strata by patient status**. The effects of age, smoking, and alcohol consumption on LINE-1 methylation levels are shown. Click here for file
