# Supplementary material for: LINE-1 methylation is inherited in familial testicular cancer kindreds
Source: BMC Med Genet. 2010 May 17;11:77. doi: 10.1186/1471-2350-11-77 (PMC2880977; doi:10.1186/1471-2350-11-77)
Supplement: Additional file 1 — Global (LINE-1) methylation levels among strata by patient status. The effects of age, smoking, and alcohol consumption on LINE-1 methylation levels are shown. [file 1471-2350-11-77-S1.DOC]

| Additional File 1: Global (LINE-1) methylation levels among strata by patient status. | | | | | | |
| --- | --- | --- | --- | --- | --- | --- |
| Strata | Healthy family | |  | TGCT patients | | *P*^†^ |
|  | No. (%) | Mean (95% CI) |  | No. (%) | Mean (95% CI) |  |
| Age |  |  |  |  |  |  |
| 0-25 | 50 (19.6) | 79.29 (78.8-79.8) |  | 11 (7.2) | 78.81 (77.6-80.0) |  |
| 26-50 | 83 (32.5) | 79.23 (78.8-79.6) |  | 109 (71.7) | 79.43 (79.0-79.8) |  |
| 51-75 | 103 (40.4) | 79.11 (78.7-79.5) |  | 31 (20.4) | 79.28 (78.5-80.0) |  |
| 76-100 | 19 (7.5) | 79.21 (78.3-80.1) |  | 1 (0.7) | 79.25 (75.2-83.3) | 0.941 |
|  |  | *r*^‡^ = -0.031 (0.625) |  |  |  |  |
| Alcohol use | |  |  |  |  |  |
| Never | 87 (43.9) | 78.77 (78.4-79.2) |  | 40 (27.4) | 78.75 (78.1-79.4) |  |
| Former | 30 (15.2) | 79.59 (78.9-80.3) |  | 33 (22.6) | 80.03 (79.2-80.7) |  |
| Current | 81 (40.9) | 78.99 (78.6-79.4) |  | 73 (50.0) | 79.39 (78.9-79.9) | 0.120 |
| Smoking status | |  |  |  |  |  |
| Never | 124 (61.7) | 78.95 (78.6-79.3) |  | 97 (66.0) | 79.11 (78.7-79.5) |  |
| Former | 47 (23.4) | 79.27 (78.7-79.8) |  | 39 (26.5) | 79.93 (79.3-80.6) |  |
| Current | 30 (14.9) | 78.95 (78.3-79.6) |  | 11 (7.5) | 79.33 (78.1-80.6) | 0.626 |
| ^†^ For methylation level difference between variable strata in healthy family, adjusted for age and/or sex;  ^‡^ Spearman rank correlation coefficient for the association between age as a continuous variable and methylation levels, adjusted for sex. | | | | | | |
